# Supplementary material for: Outer membrane vesicles from a mosquito commensal mediate targeted killing of Plasmodium parasites via the phosphatidylcholine scavenging pathway
Source: Nat Commun. 2023 Aug 24;14:5157. doi: 10.1038/s41467-023-40887-6 (PMC10449815; doi:10.1038/s41467-023-40887-6)

Supplementary Information

Outer membrane vesicles from a mosquito commensal mediate targeted killing of *Plasmodium* parasites via the phosphatidylcholine scavenging pathway

Han Gao[1,2†], Yongmao Jiang[1,2†], Lihua Wang[1,2†], Guandong Wang[1,2], Wenqian Hu[1,2], Ling Dong[1,2], Sibao Wang[1,2*]

1 CAS Key Laboratory of Insect Developmental and Evolutionary Biology, CAS Center for Excellence in Molecular Plant Sciences, Shanghai Institute of Plant Physiology and Ecology, Chinese Academy of Sciences, Shanghai, China.

2 CAS Center for Excellence in Biotic Interactions, University of Chinese Academy of Sciences, Beijing, China.

*** To whom corresponding may be addressed:**

Wang, S., CAS Center for Excellence in Molecular Plant Sciences, Shanghai Institute of Plant Physiology and Ecology, Chinese Academy of Sciences

300 Feng Lin Road, Shanghai, China

E-mail: sbwang@cemps.ac.cn

Phone: +86 (0)21-54924339; FAX: +86 (0)21-54924339

Supplementary figures and legends

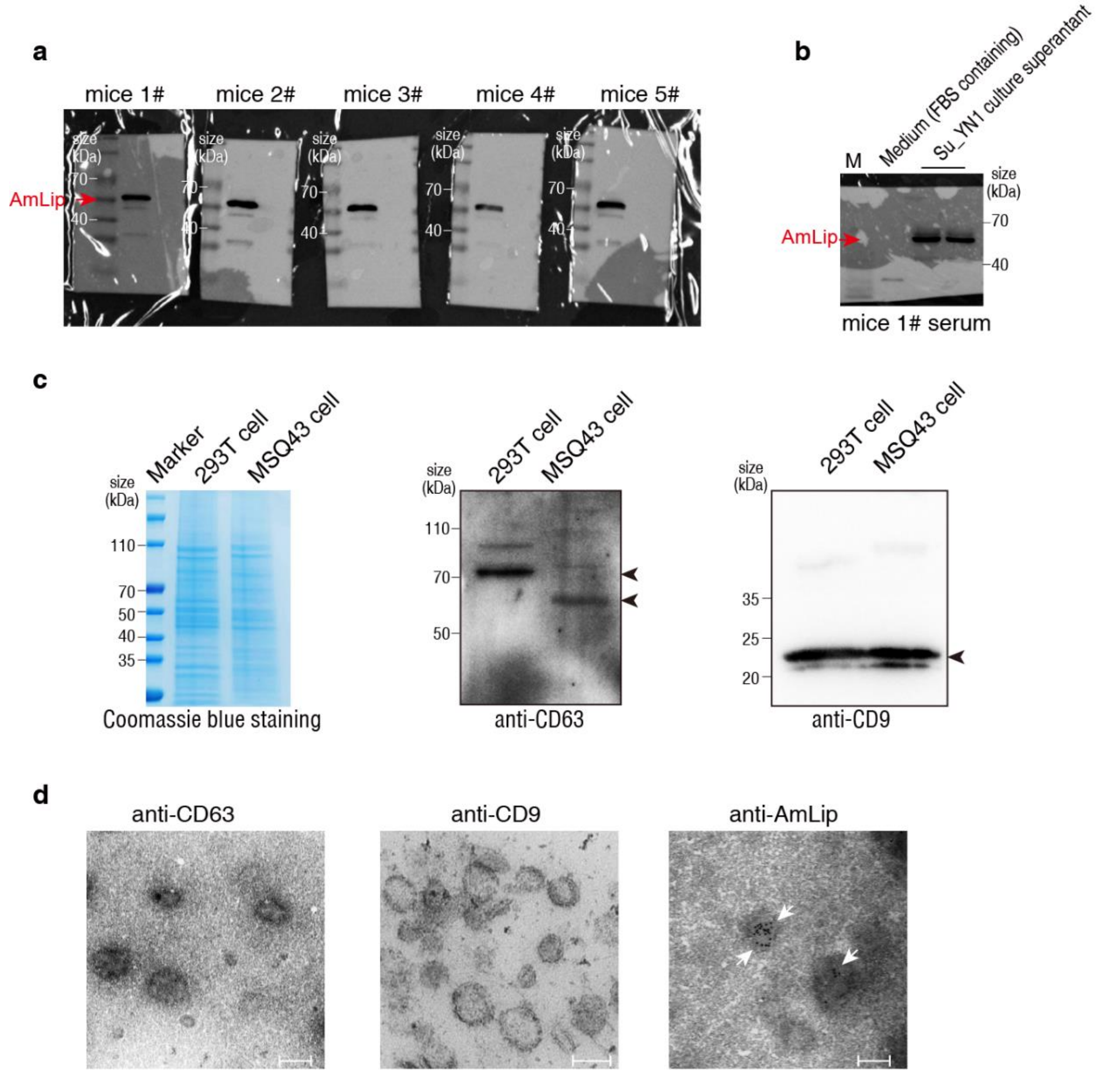

Supplementary Figure 1 | Specificity test of AmLip antiserum and eukaryotic exosome markers.

a, Western blot of Su_YN1 culture supernatant using AmLip mouse antiserum. Antiserums from five mice immuned with AmLip antigen were tested for their specificity. The images are Western blot signals merged with original PVDF membrane. Red arrows indicate AmLip protein bands detected. Similar results were obtained from two biological repeats. **b,** Western blot of AmLip protein in the control culture medium (RPMI 1640 medium with 10% FBS) and Su_YN1 culture supernatant. The AmLip antiserum (from mice 1#) showed a clear background in the medium lane. The images are Western blot signals merged with original PVDF

membrane. Red arrows indicate AmLip protein bands detected. Similar results were obtained from two biological repeats. **c,** Western blot detection of CD63 and CD9 using 30 μg of total protein extracts obtained from *Anopheles* MSQ43 cells and human 293T cells. The left panel shows Commassie blue staining of the samples as a loading control. The arrowheads indicate the target bands. Similar results were obtained from two biological repeats. **d,** Immunoelectron microscopy (IEM) detection of CD63, CD9 and the lipase AmLip on OMVs in an ultrathin cryosection of the midgut of Su_YN1-carrying *Anopheles* mosquito. Positive staining is indicated by white arrowheads. The scale bar, 100 nm. Similar results were obtained from two biological repeats.

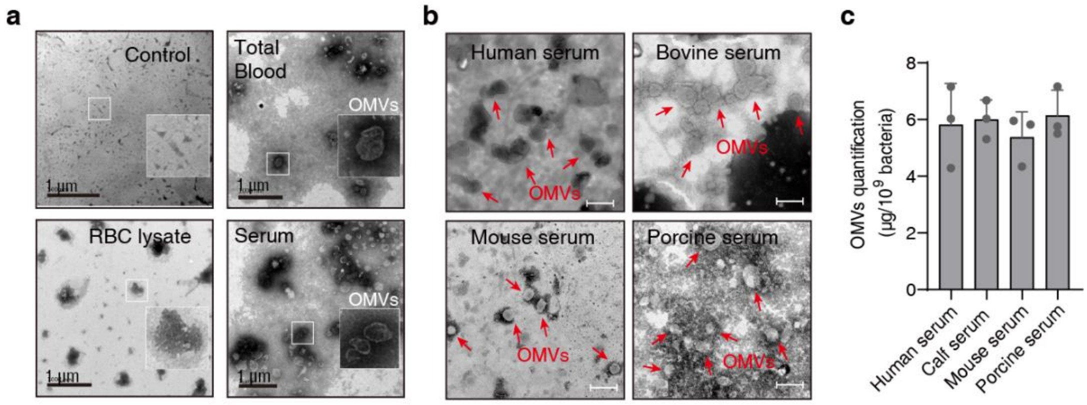

d

Sample	particle size	Particle concentration	Vesicle portion
Human serum mock purified	Unable to detect.		
**Human serum induced OMVs**	**82.50±15.85 nm**	**2.78E+11 / mL**	**99.05%**
Bovine serum mock purified	Unable to detect.		
**Bovine serum induced OMVs**	**82.37±14.47 nm**	**3.29E+11 / mL**	**98.44%**
Mouse serum mock purified	Unable to detect.		
**Mouse serum induced OMVs**	**80.06±14.93 nm**	**1.86E+11 / mL**	**86.78%**
Porcine serum mock purified	80.76±16.70	1.21E+08 / mL	86.49%
**Porcine serum induced OMVs**	**82.00±15.29 nm**	**1.66E+11 / mL**	**89.28%**

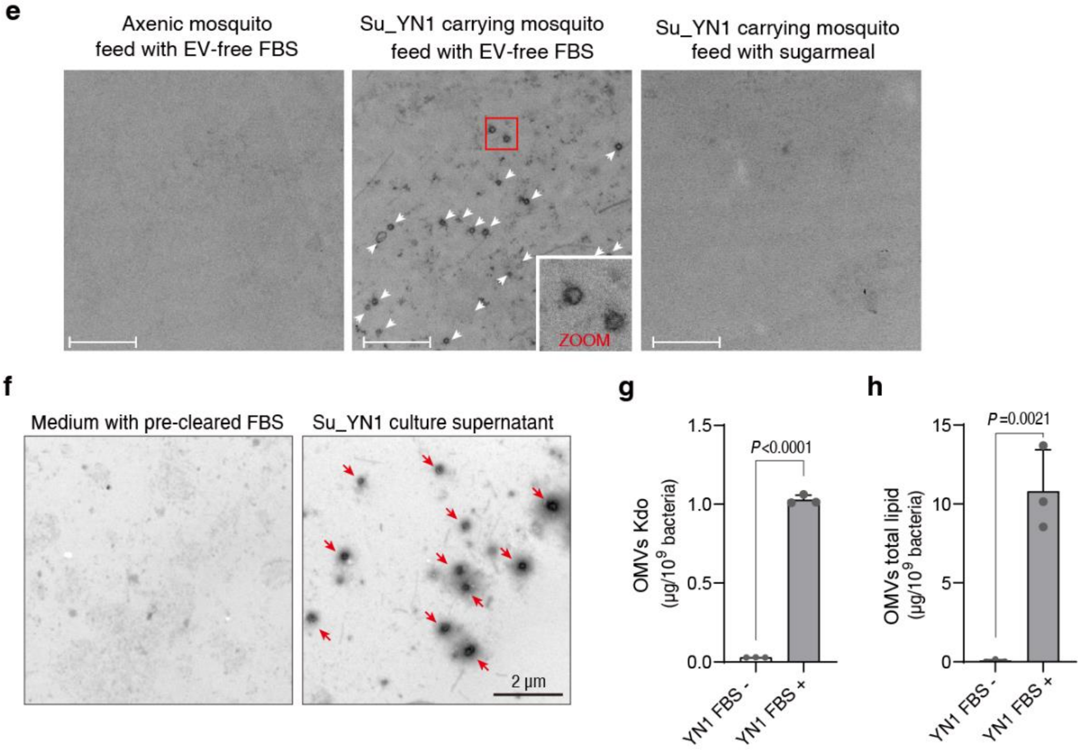

Supplementary Figure 2 | Host serum induces OMV production.

a, TEM observation of OMV production in the supernatant of Su_YN1 cultured with different blood components. Total blood, red blood cell (RBC) lysate and serum were used to stimulate OMV production of Su_YN1 in RPMI 1640-based medium. Control

is supernatant of RPMI 1640 containing total blood cultured without bacterium. Scale bar, 100 nm. Similar results were obtained from two biological repeats. **b,** TEM observation of OMVs from Su_YN1 supernatant cultured in RPMI 1640 medium containing 10% extracellular vesicles-depleted human serum, bovine serum, mouse serum or porcine serum. Red arrowheads point to OMVs. Scale bar, 200 nm. **c,** OMV quantification of Su_YN1 cultured with various sera using the bicinchoninic acid (BCA) assay (mean $\pm$SD, n=3). OMV concentrations are normalized to 1×10^9 bacteria. Statistical significance was determined using one-way ANOVA. The *P* value indicates comparisons with the control group. Similar results were obtained from two biological repeats. **d,** Nanoflow cytometry measurement (NFCM) of purified OMVs from Su_YN1 culture with serum from various sources. Mock-purified mediums were used to demonstrated background vesicles. **e,** TEM observation of Su_YN1 OMVs from the mosquito midgut lumen, that *Anopheles* mosquitoes carrying Su_YN1 or axenic *Anopheles* mosquitoes were feed with FBS or sugar meal. The white arrow heads indicate OMVs. Scale bar, 1 μm. Similar results were obtained from two biological repeats. **f,** TEM observation of OMVs in the RPMI 1640 medium containing 10% pre-cleared FBS or Su_YN1 culture supernatant. Red arrows indicate OMVs. Scale bar, 2 μm. **g,** OMV quantification of Su_YN1 cultured with or without FBS using the 3-Deoxy-d-manno-octulosonic acid assay (mean $\pm$SD, n=3). Statistical significance was determined using a two-tailed Student's *t*-test, *P* values are indicated above the plots. **h,** OMV quantification of Su_YN1 cultured with or without FBS using a total lipid assay (mean $\pm$SD, n=3). Statistical significance was determined using a two-tailed Student's *t*-test, *P* values are indicated above the plots. The OMV quantifications in (g) and (h) are normalized to 1×10^9 bacteria, and similar results were obtained from two biological repeats. Source data are provided as Source Data File.

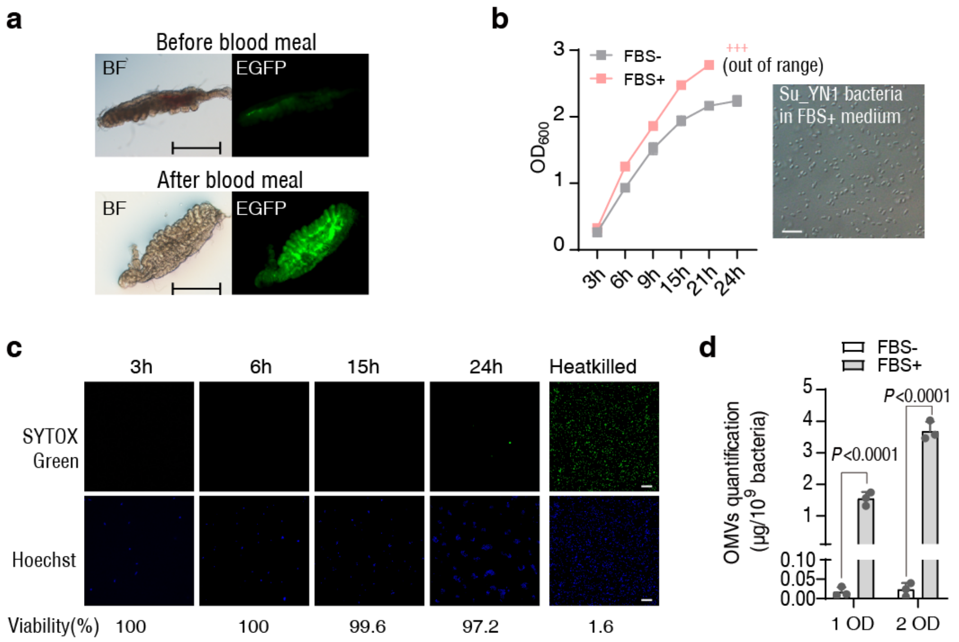

Supplementary Figure 3 | The viability test of *Serratia ureilytica* Su_YN1.

a, Mosquito midgut carrying eGFP-expressing Su_YN1 bacterium before and 48 h post a blood meal. BF, bright field. Scale bar, 1 mm. **b,** Growth curve of Su_YN1 cultured in RPMI 1640 medium with or without 10% FBS. +++ means the bacterium density exceeds the detection limit of the spectrophotometer. Microphotograph in the right panel indicates living Su_YN1 bacteria cultured in RPMI 1640 medium with 10% FBS. The bacteria show elliptical shape and swim actively in the medium. Scale bar, 10 μm. Similar results were obtained from three biological repeats. **c,** Viability test of Su_YN1 bacteria cultured in RPMI 1640 medium with 10% FBS using Hoechst/ SYTOX Green staining method. The Hoechst signal (blue) indicates all the bacteria, while the SYTOX Green signal (green) indicates dead bacteria. Heat-killed indicates Su_YN1 bacteria pre-treated at 95°C for 5 min to kill the bacteria. Viability means the proportion of living bacteria (SYTOX Green) of all the bacteria (Hoechst). Scale bar, 10 μm. **d,** Quantification of OMVs of Su_YN1 cultured under FBS- and FBS+ conditions once the bacterial density reaches 1OD and 2OD in each group, respectively. The results are presented as mean ± SD (n=3). OMV concentrations are normalized to 1×10^9 bacteria. Statistical significance was determined using a two-tailed Student's *t*-test, *P* values are indicated above the plots. Source data are provided as a Source Data File.

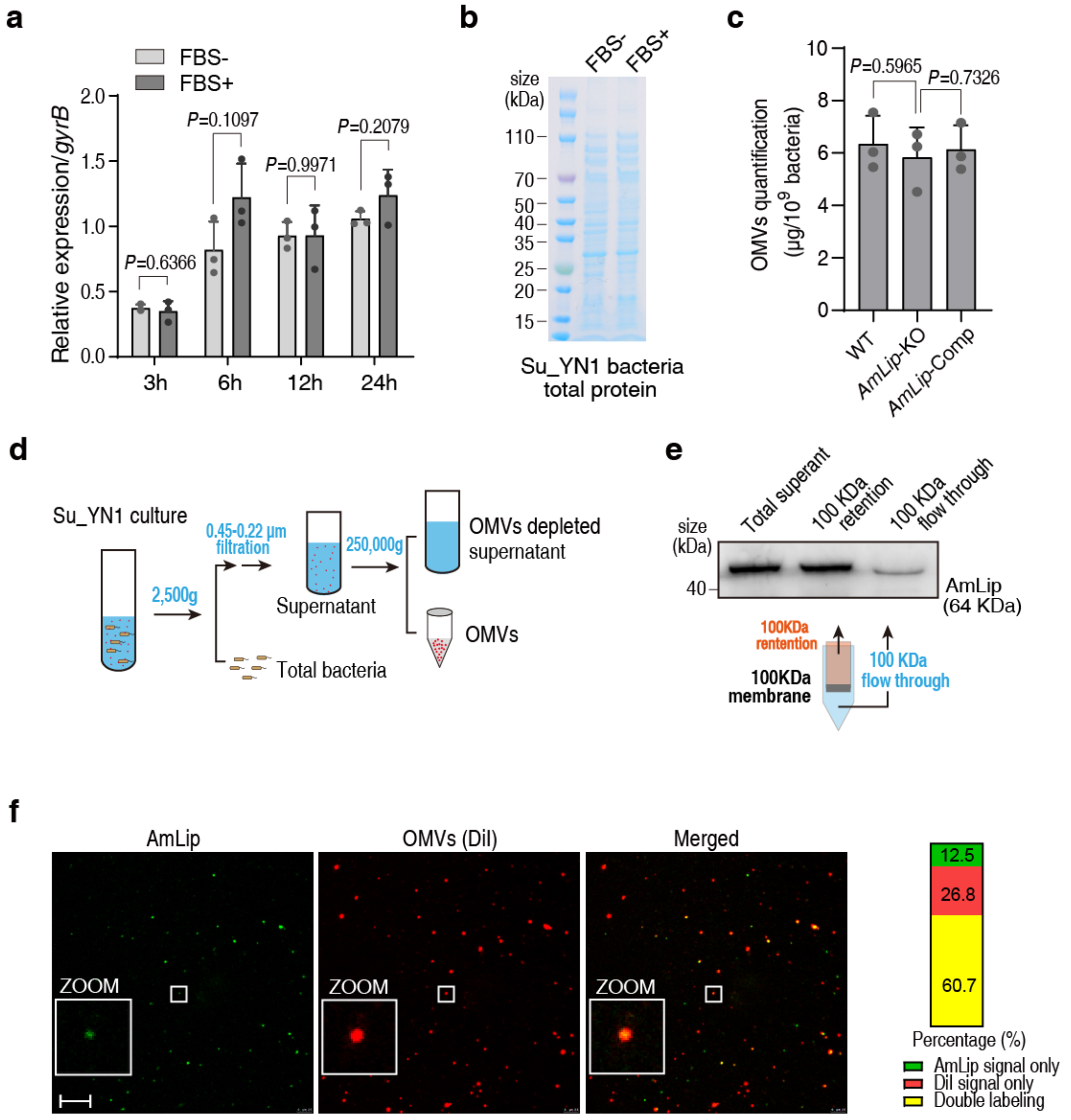

Supplementary Figure 4 | AmLip transcription and protein expression analysis. **a,** Transcription analysis of *AmLip* in Su_YN1 cultured in RPMI 1640 medium with or without 10% FBS using qPCR. The *Serratia* stably expressed gene *gyrB* was used as a reference gene. Statistical significance was determined using a two-tailed Student's *t*-test (mean ± SD, n=3). *P* values are indicated above the plots. **b,** Commissive blue staining of total Su_YN1 protein equally extracted from 1 OD bacteria. **c,** OMV quantification in Su_YN1 wildtype (WT), its AmLip-knockout (*AmLip*-KO) mutant and *AmLip*-complemented strains using BCA assay (mean ± SD, n=3). OMV concentrations are normalized to 1×10^9 bacteria. Statistical significance was determined using one-way ANOVA, *P* values are indicated above the plots. **d,**

Diagram showing Su_YN1 OMV fraction analysis, corresponding to Fig.2(a) and (b). **e,** Size fraction assay of AmLip distribution in Su_YN1 culture supernatant by 100 KDa ultrafiltration. 20 ul of each fraction was used for detection. Distribution of AmLip was detected by Western blot using AmLip antiserum. Similar results were obtained from two biological repeats. **f,** Confocal images showing colocalization of AmLip (green channel) and OMVs (DiI stained, red channel). The right panel shows quantification of the signals. Scale bar, 5 μm. Similar results were obtained from two biological repeats. Source data are provided as a Source Data File.

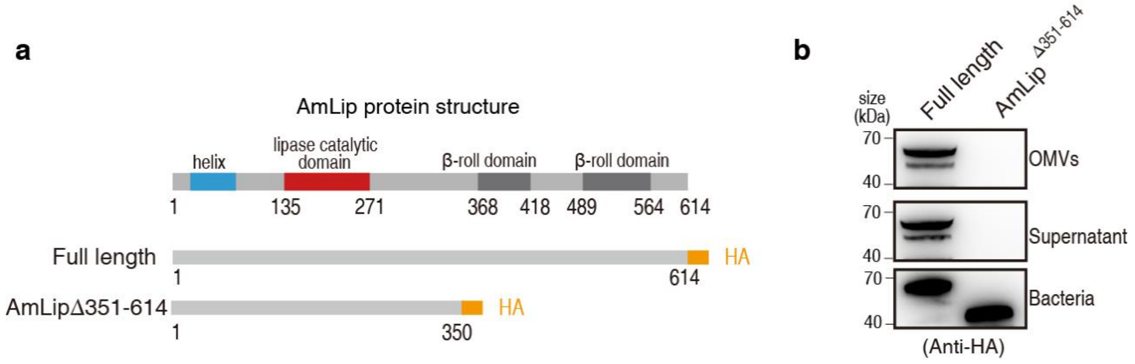

Supplementary Figure 5 | AmLip secretion via T1SS is critical for its binding to OMVs.

a, Diagram showing AmLip protein structure and truncated constructs. C-terminal of the constructs was fused with Influenza Hemagglutinin peptide (HA). **b,** Western blot analysis of AmLip constructs using an anti-HA antibody. The distribution of AmLip in bacterial cell (20 μg of total bacterial protein), culture supernatant (20 ul of culture supernatant), and OMVs (20 μg) was assayed. Similar results were obtained from two biological repeats.

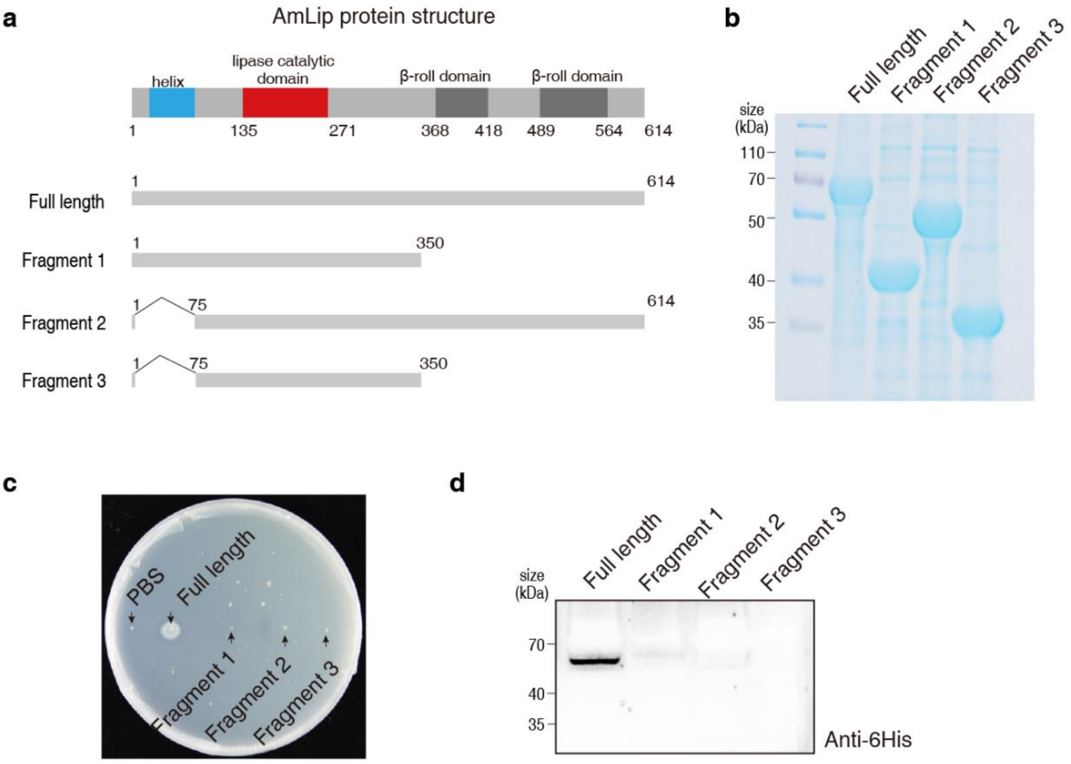

Supplementary Figure 6 | OMV binding assay of AmLip protein and AmLip protein fragments.

a, Schematic diagram of AmLip protein structure and AmLip fragments. The fragment 1 lacks the C-terminal (Δ351-614), which is required for recognition by T1SS, fragment 2 lacks the N-terminal lid structure (Δ1-74), while fragment 3 lacks both the C-terminal and N-terminal regions (Δ1-74, Δ351-614). **b,** Coomassie blue staining of recombinantly expressed 6×His-tagged AmLip protein and AmLip fragments. **c,** Lipase activity test of purified AmLip protein and protein fragments on egg yolk plate. **d,** Western blot analysis of AmLip protein and AmLip protein fragments bound to OMVs using anti-6×His Tag antibody. Similar results were obtained from two biological repeats.

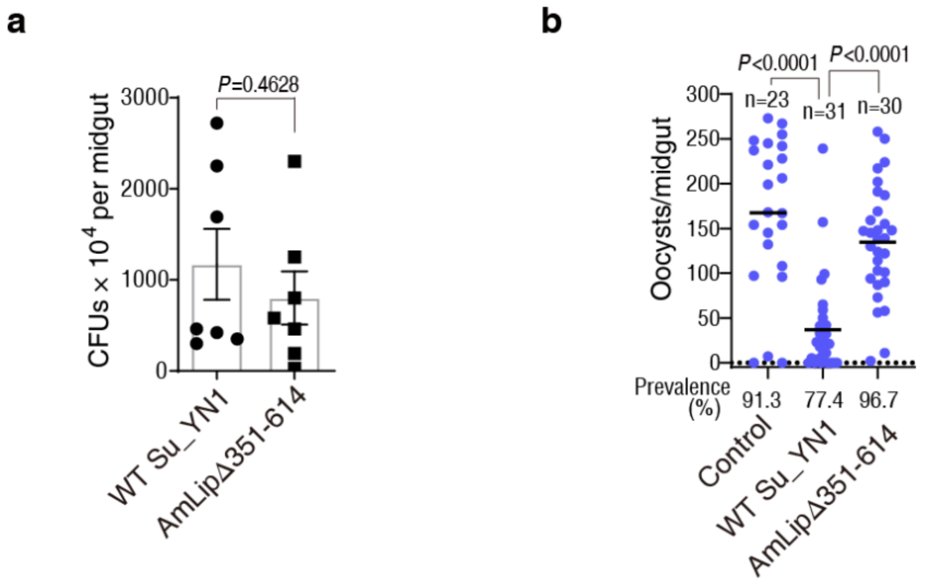

Supplementary Figure 7 | Gut colonization and *Plasmodium* blocking assay of wildtype Su_YN1 strain and AmLip C-terminal T1SS-deleted mutant strain.

a, Gut colonization assay of wildtype (WT) Su_YN1 strain and AmLip C-terminal T1SS-deleted mutant (AmLip Δ351-614) strain in *An. stephensi*. Colony forming units (CFU) were counted from isolated midguts (mean ± SD, n=7) of the bacteria-carrying mosquitoes 48 h post blood feeding. The statistical significance of the CFUs between the groups was analyzed using the two-tailed Mann-Whitney test, *P* values are indicated above the plots. Similar results were obtained from two biological repeats. **b,** Pb ANKA oocyst load in the midgut of *An. stephensi* mosquitoes fed with WT Su_YN1 strain, AmLip Δ351-614 mutant strain, or PBS (Control). Circles represent the number of oocysts in individual midguts and horizontal lines indicate the median number of oocysts per midgut. The statistical significance of the oocyst intensity between the groups was analyzed using the two-tailed Mann-Whitney test, *P* values are indicated above the plots. Similar results were obtained from two biological repeats. Source data are provided as a Source Data File.

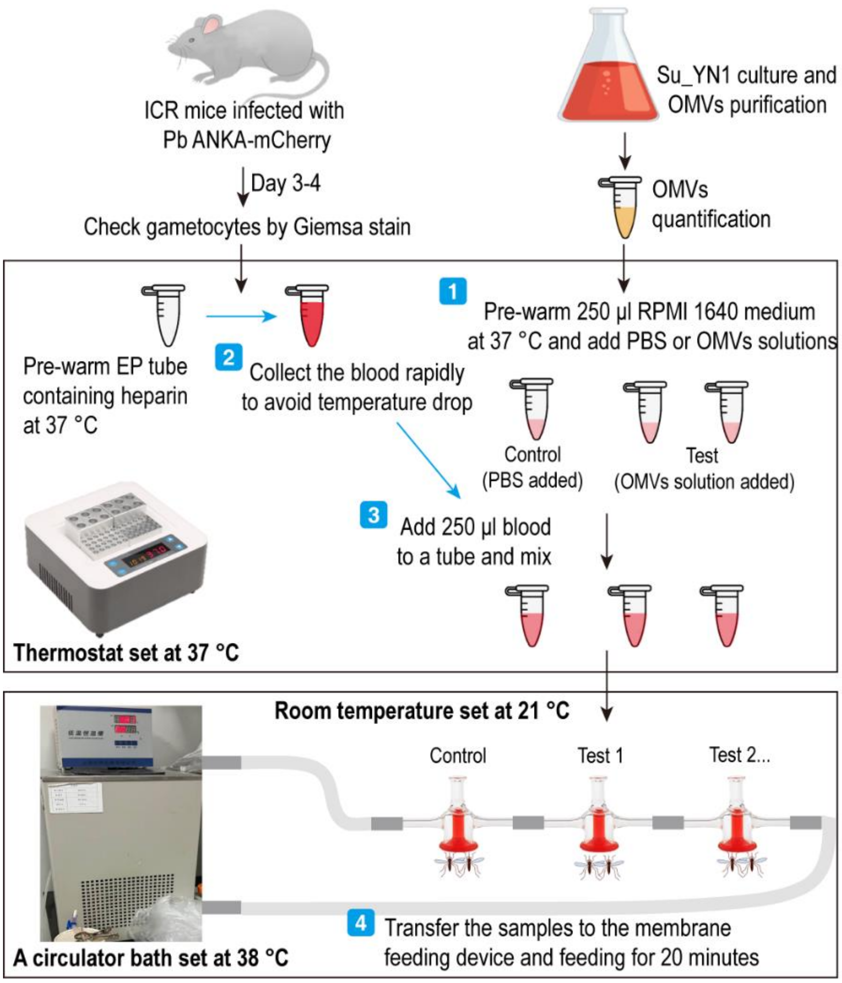

Supplementary Figure 8 | Workflow of *P. berghei* Standard Membrane Feeding Assays (Pb SMFAs).

Schematic representation of important procedures to carry out a successful Pb SMFAs. Mice infected with Pb ANKA mCherry strain with high gametocytemia at day 3-4 post-infection were used for collecting infectious blood. When decide to carry out the feeding, 1) set up and pre-warm the membrane feeding devices, as well as tubes containing the samples to be tested (control PBS and OMVs solution added in RPMI 1640) in a dry thermostat to 37 °C; 2) we prewarmed the tube (containing heparin to collect blood) in a dry thermostat to 37 °C, and collected mouse blood rapidly in the pre-warmed tube; 3) immediately, transfer the blood to the sample-containing tube, mix and instantly add to the membrane feeding device connected to a circulator bath set at 38 °C for feeding; 4) carry out feeding in a room with environmental temperature set at 21 °C. Feeding lasts for 15-20 min.

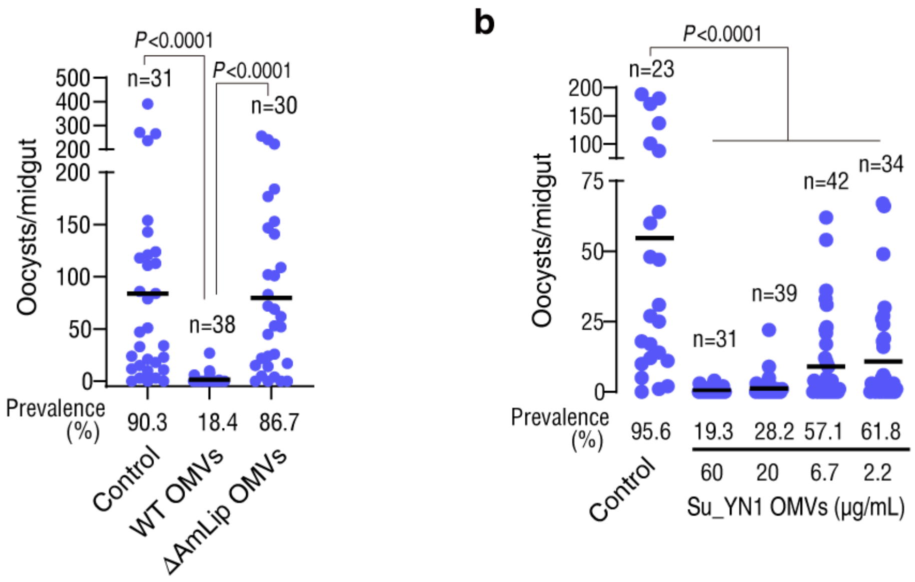

Supplementary Figure 9 | AmLip-containing Su_YN1 OMVs kill *Plasmodium* parasites in the mosquito midgut.

a, Effect of wildtype Su_YN1 OMVs and *AmLip* gene disrupted mutant (Δ*AmLip*) OMVs on oocyst formation in the mosquito midgut by Pb ANKA Standard Membrane Feeding Assays (Pb SMFAs). Final concentration of 50 μg/ml OMVs were added to the infectious blood for feeding *An. stephensi* mosquitoes. PBS was used as a Control. Oocyst load is shown as circles in individual midguts and horizontal lines indicate the median number of oocysts per midgut. The statistical significance of the oocyst intensity between the groups was analyzed using the two-tailed Mann-Whitney test. *P* values are indicated above the plots. Similar results were obtained from two biological repeats. **b,** Inhibitory effect of different amounts of Su_YN1 OMVs on oocyst formation in the mosquito midgut via Pb SMFAs. Oocyst load is shown as circles in individual midguts, and horizontal lines indicate the median number of oocysts per midgut. The statistical significance of the oocyst intensity between the control groups (PBS) and OMVs-added groups was analyzed using the two-tailed Mann-Whitney test.

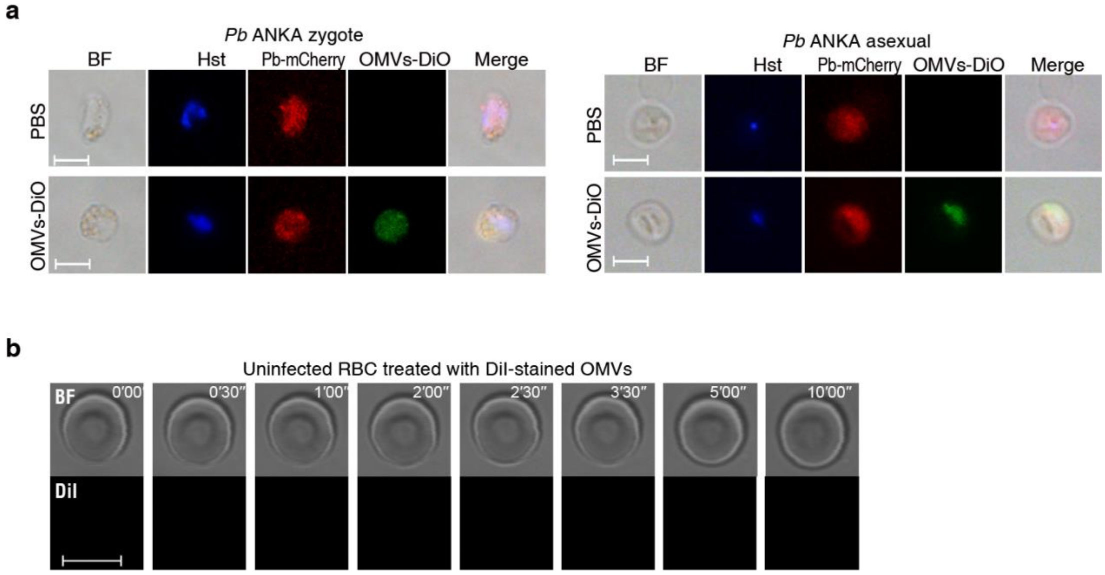

Supplementary Figure 10 | Su_YN1 OMVs rapidly enter *Plasmodium* parasites. **a,** Fluorescence microscope observation of *P. berghei* ANKA-mCherry parasites incubated with DiO-labeled (green) OMVs. Zygote (left panel) and asexual (right panel) parasites were incubated with DiO-labeled OMVs for 1 h prior to observation. Nucleus of parasite was stained with Hoechst 33342 (Hst). BF, bright field. Scale bar, 5 µm. Similar results were obtained from two biological repeats. **b,** Confocal live tracking of DiI-labeled OMV uptake by uninfected red blood cell (RBC). Images were captured at 30 sec intervals for a duration of 10 min following the addition of DiI-labeled OMVs to the RBC. BF, bright field. Scale bar, 5 µm. Similar results were obtained from two biological repeats.

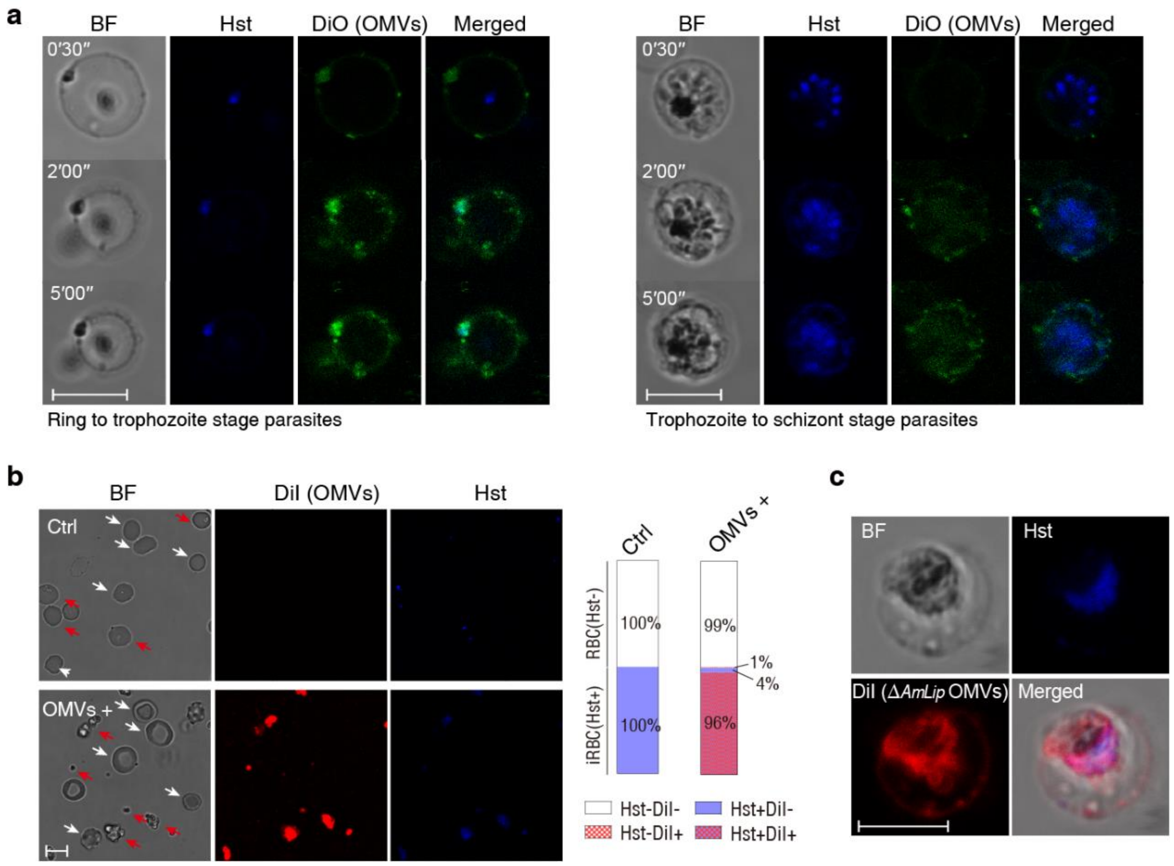

Supplementary Figure 11 | Su_YN1 OMVs selectively enter and kill *Plasmodium falciparum* asexual stages.

a, Confocal live tracking of *P. falciparum* 3D7 asexual parasites incubated with DiO-labeled OMVs. The left panel is a ring stage parasite, and the right panel is a schizont stage parasite. Images were captured at various times, as labeled. Parasite nuclei were stained with Hoechst 33342 (Hst). BF, bright field. Scale bar, 5 µm. Similar results were obtained from two biological repeats. **b,** Confocal image of *P. falciparum* 3D7 asexual parasites incubated for 20 min with DiI-labeled OMVs. The white arrowheads indicate healthy red blood cells (RBCs), the red arrowheads indicate parasitized cells that were lysed by Su_YN1 OMVs. Parasite nuclei were stained with Hoechst 33342 (Hst). BF, bright field. Scale bar, 5 µm. The right panel represents statistical analysis of the DiI signal distribution in RBCs (Hst negative, Hst-) and iRBC (Hst positive, Hst+). Similar results were obtained from two biological repeats. **c,** Confocal image of *P. falciparum* 3D7 asexual parasites incubated with DiI-stained OMVs from the AmLip-KO mutant strain. Parasite nuclei were stained with Hoechst 33342 (Hst). BF, bright field. Scale bar, 5 µm. Similar results were obtained from two biological repeats.

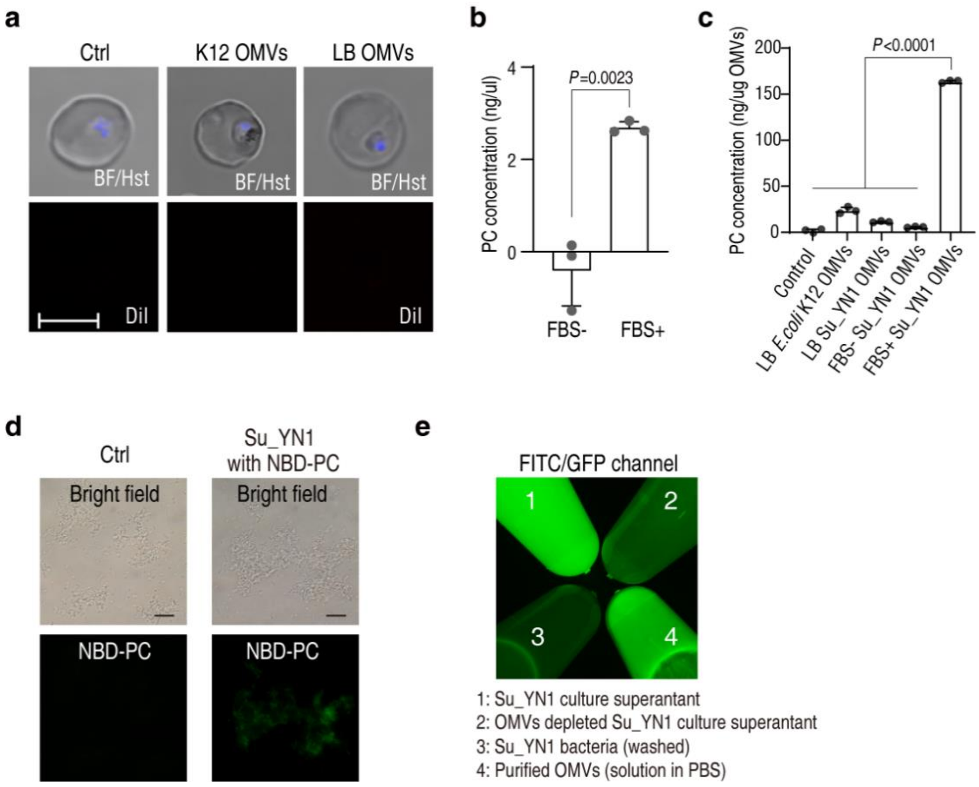

Supplementary Figure 12 | Phosphatidylcholine is efficiently incorporated into Su_YN1 OMVs.

a, Uptake of OMVs from different bacteria cultured in Luria Broth (LB) medium by asexual *P. falciparum* 3D7 parasites. OMVs from *E. coli* K12 and *Serratia* Su_YN1 cultured in LB were named K12 OMVs and LB OMVs. *P. falciparum* 3D7 asexual parasites were incubated DiI-stained OMVs and OMV uptake was followed by monitoring DiI signals on parasite cells. Control (Ctrl) means parasites treated with PBS. The parasite nucleus was stained with Hoechst 33342 (Hst). BF, bright field. Scale bar, 5 µm. **b,** Phosphatidylcholine quantification of *Serratia* Su_YN1 grown in RPMI 1640 medium (FBS-) or RPMI 1640 medium supplemented with 10% FBS (FBS+). Equal amounts of bacteria (1 OD) were used for extraction and detection (mean ± SD, n=3). Statistical significance was determined using a two-tailed Student's *t*-test, *P* value is indicated above the plots. **c,** Phosphatidylcholine quantification assay of OMVs from various sources. OMVs from *E. coli* K12 (LB *E. coli* K12 OMVs) and *Serratia* Su_YN1 (LB Su_YN1 OMVs) cultured in LB, or *Serratia* Su_YN1 cultured in RPMI 1640 with (FBS+ Su_YN1 OMVs) or without

FBS (FBS- Su_YN1 OMVs) were tested. The PBS control lane was prepared by mock purifying and washing RPMI 1640 plus 10% EV-free FBS+ medium using the same OMV purification procedure by ultracentrifugation that was used for the OMVs. 100 μg OMVs were used for PC quantification (mean ± SD, n=3). Statistical significance was determined using one-way ANOVA test, *P* value is indicated above the plots. Similar results were obtained from two biological repeats. **d,** Fluorescence images of Su_YN1 bacteria cultured with or without 3mM Nitrobenzoxadiazole labeled Phosphatidylcholine (NBD-PC). Scale bar, 5 μm. Similar results were obtained from two biological repeats. **e,** Detection of NBD-PC incorporation into *Serratia* Su_YN1 and bacterial culture fractions. Su_YN1 culture supernatant (1), OMVs-depleted Su_YN1 culture supernatant (2), Su_YN1 bacteria (3) and OMV solution in tubes (4) was checked directly using FITC optical filter to detect the presence of NBD-PC signals. Source data are provided as a Source Data File.

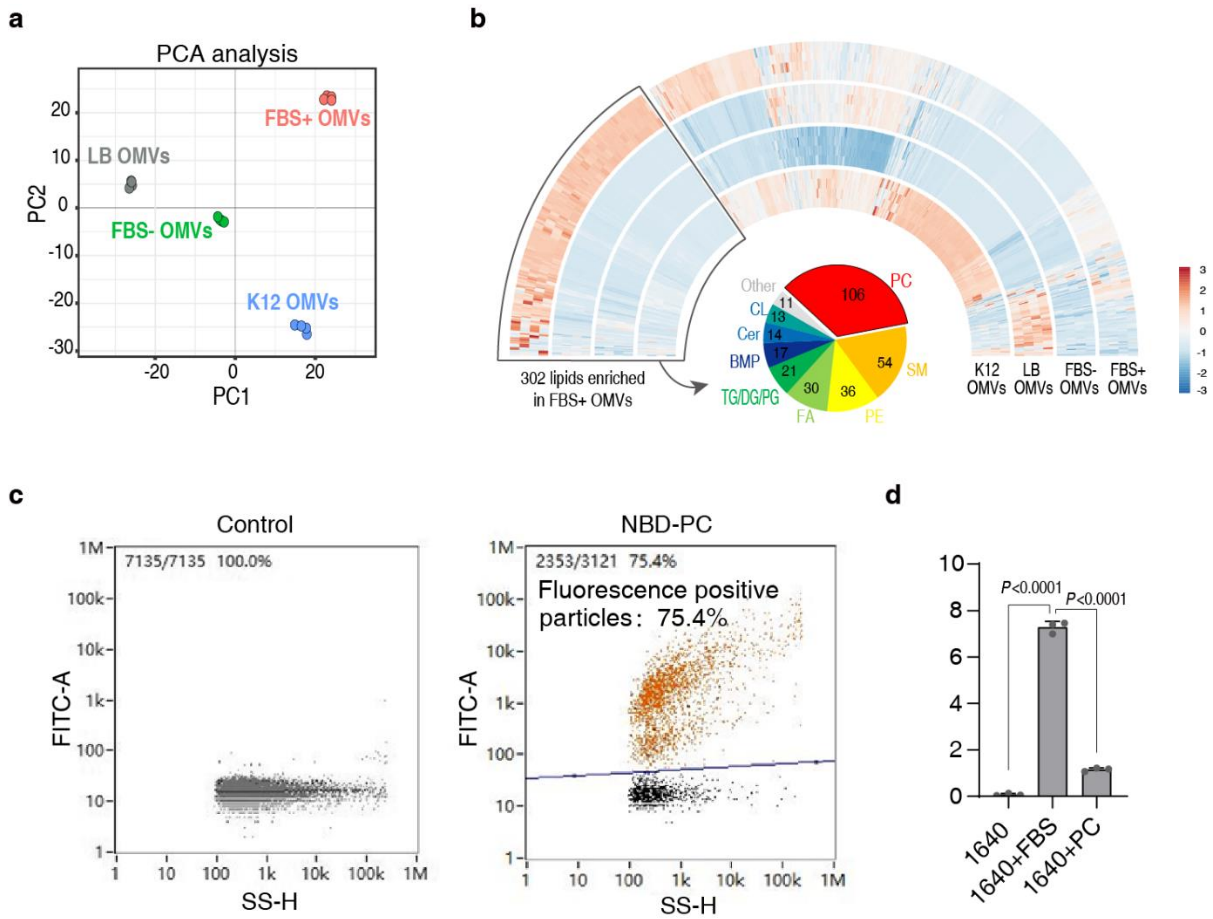

Supplementary Figure 13 | Comparative lipidome analysis of OMVs from various resources.

a, Principal component analysis (PCA) of the lipid profiles of OMVs. Su_YN1 FBS+ OMVs (cultured in RPMI 1640 with FBS), FBS- OMVs (cultured in RPMI 1640 without FBS), Su_YN1 LB OMVs (cultured in LB) and *E. coli* K12 OMVs (cultured in LB) were purified, and total lipids were extracted for lipidome analysis. PCA analysis indicates significant difference among lipid profiles of these OMVs. **b,** Clustering analysis of lipid contents of FBS+ OMVs, FBS- OMVs LB OMVs and K12 OMVs. The individual rows indicate lipids detected and the individual columns indicate independent samples (n = 4 independent repeats in each group) extracted and tested by MS using Q Exactive. Specific enriched lipid clusters in FBS + OMVs are delimited with a square frame. The lipidome data was processed using MS-DIAL software (ver.4.38). The processed data was further analyzed by the Clustvis. Proportions of lipid classes in the cluster are displayed as a pie chart. **c,** Nanoflow cytometry measurement (NFCM) of the fluorescence intensity of OMVs purified from Su_YN1 cultured

without NBD-PC (Control), and OMVs purified from Su_YN1 cultured with 3mM NBD-PC supplementation. **d,** Quantification of OMVs purified from Su_YN1 cultured in RPMI 1640 medium, RPMI 1640 medium supplemented with 10% FBS, or RPMI 1640 medium supplemented with 20 μg/ml PC (to mimic physiological PC levels present in serum). The quantification of OMVs was normalized to 10^9 bacteria (mean ± SD, n=3). Statistical significance was determined using one-way ANOVA test, *P* values are indicated above the plots. Source data are provided as a Source Data File.

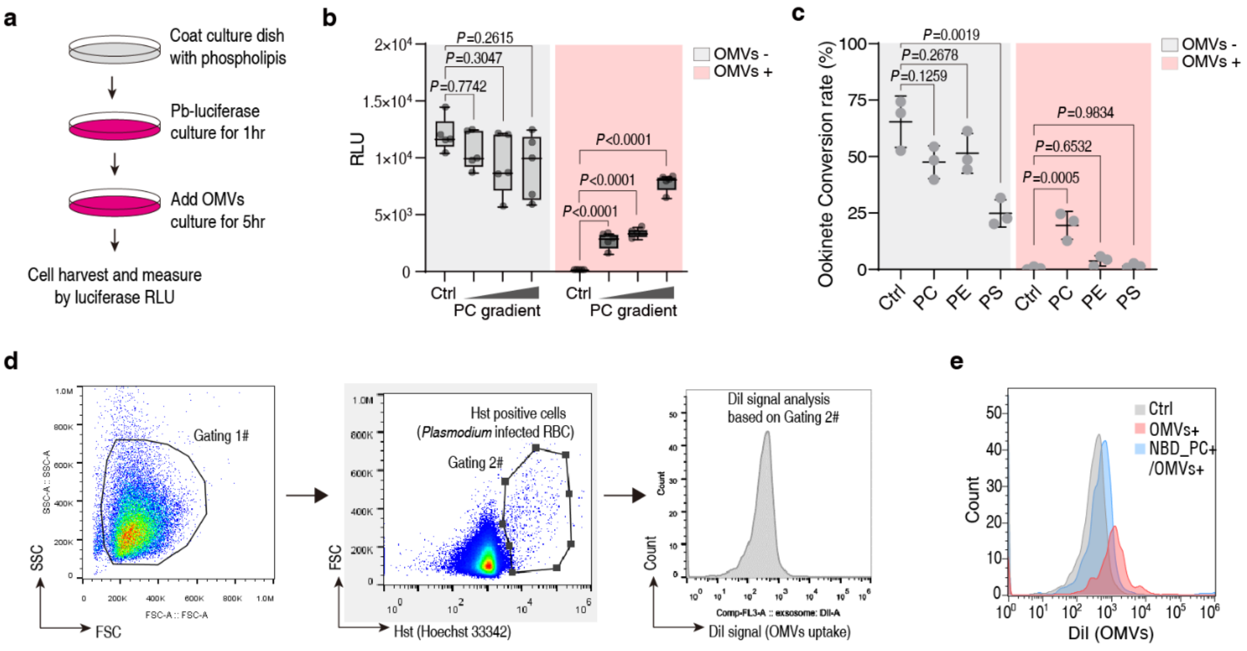

Supplementary Figure 14 | Phosphatidylcholine strongly antagonizes the OMV killing effect of ookinetes and asexual *Plasmodium* parasites

a, Diagram showing phospholipid inhibition assay of OMV antimalarial activity. Culture dishes were coated with different phospholipids, parasites were cultured in these pre-coated dishes prior to addition of OMVs. Parasite viability was measured by luciferase relative light units (RLU). **b,** *Plasmodium* viability assay of asexual *P. berghei* ANKA luciferase parasites incubated with or without Su_YN1 OMVs, and cultured in dishes coated with different PC concentrations. The PC gradients (from low to high) are 156 μg/cm^2, 312 μg/cm^2 and 625 μg/cm^2. Parasite viability (n=5) was measured by luciferase relative light units (RLU). The middle line of box-plot diagram represents median, boxes extend from the 25th to 75th percentiles. The whiskers mark the 10th and 90th percentiles. Statistical significance was determined using one-way ANOVA test, *P* values are indicated above the plots. **c,** Effects of different phospholipids on inhibition of *P. berghei* ANKA ookinete development by OMVs. Ookinetes (6 h after imitation) were cultured in dishes pre-coated with 625 μg/cm^2 phospholipids for 2 h prior to adding Su_YN1 OMVs at a final concentration of 100 μg/ml and cultured for an additional 3 h. Ookinete conversation rates were calculated (mean ± SD, n=3). Evaporated chloroform solvent was used as a control (Ctrl). Similar results were obtained from two biological repeats. Statistical significance was determined using one-way

ANOVA test, *P* values are indicated above the plots. **d,** Sequential gating strategy for OMVs uptake by *P. falciparum* 3D7 asexual stage parasites. Gated out parasites (Hoechst 33342 positive cell populations, Gating 2#) were further analyzed for DiI signal. **e,** Flow cytometry analysis of antagonistic effects of PC on OMV uptake of *Plasmodium* parasites. *P. falciparum* 3D7 asexual stage parasites were incubated with NBD-PC for 10 min before incubation with DiI-stained Su_YN1 OMVs for another 2 min followed by flow cytometry. The uptake of OMVs (DiI signal) by parasite cell population (Hst positive, Gating 2# in figure d) is displayed as histogram. Similar results were obtained from two biological repeats. Source data are provided as a Source Data File.

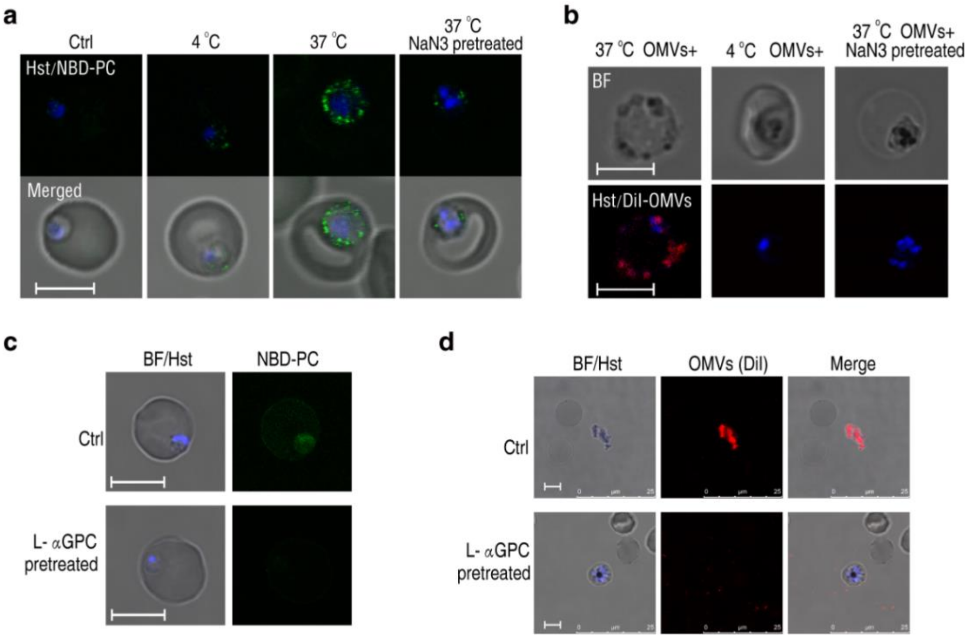

Supplementary Figure 15 | Manipulating phosphatidylcholine scavenge pathway in OMV uptake of the *Plasmodium parasite*.

a, Fluorescence images of uptake of nitrobenzoxadiazole labeled phosphatidylcholine (NBD-PC) by *P. falciparum* 3D7 asexual parasites treated under various conditions for inhibiting PC scavenge pathway. NaN3: sodium azide. Control (Ctrl) means parasites treated with solvent. Parasite nuclei were stained with Hoechst 33342 (Hst). BF, bright field. Scale bar, 5 µm. Similar results were obtained from two biological repeats. **b,** *P. falciparum* 3D7 asexual stage parasites were pre-treated under various conditions (at different temperature or pretreated with sodium azide (NaN3) to deplete ATP) prior to incubation for 5 min with DiI-stained Su_YN1 OMVs. Uptake of OMVs is indicated by the DiI fluorescent signal on parasite cells. Scale bar, 5 µm. Similar results were obtained from two biological repeats. **c,** Fluorescence images of NBD-PC uptake by *P. falciparum* 3D7 asexual parasites pre-treated with 5mM L-α Glycerylphosphorylcholine (L-α GPC). Control (Ctrl) means parasites treated with solvent. Parasite nuclei were stained with Hoechst 33342 (Hst). BF, bright field. Scale bar, 5 µm. Similar results were obtained from two biological repeats. **d,** *P. falciparum* 3D7 asexual stage parasites were pre-treated with 5mM L-α-GPC to inhibit the PC scavenge pathway prior to incubation for 5 min with DiI-stained Su_YN1 OMVs. Uptake of OMVs is indicated by the DiI fluorescent signal on parasites. Parasite nuclei were stained with Hoechst 33342 (Hst). BF, bright field. Scale bar, 5 µm. Similar results were obtained from two biological repeats.

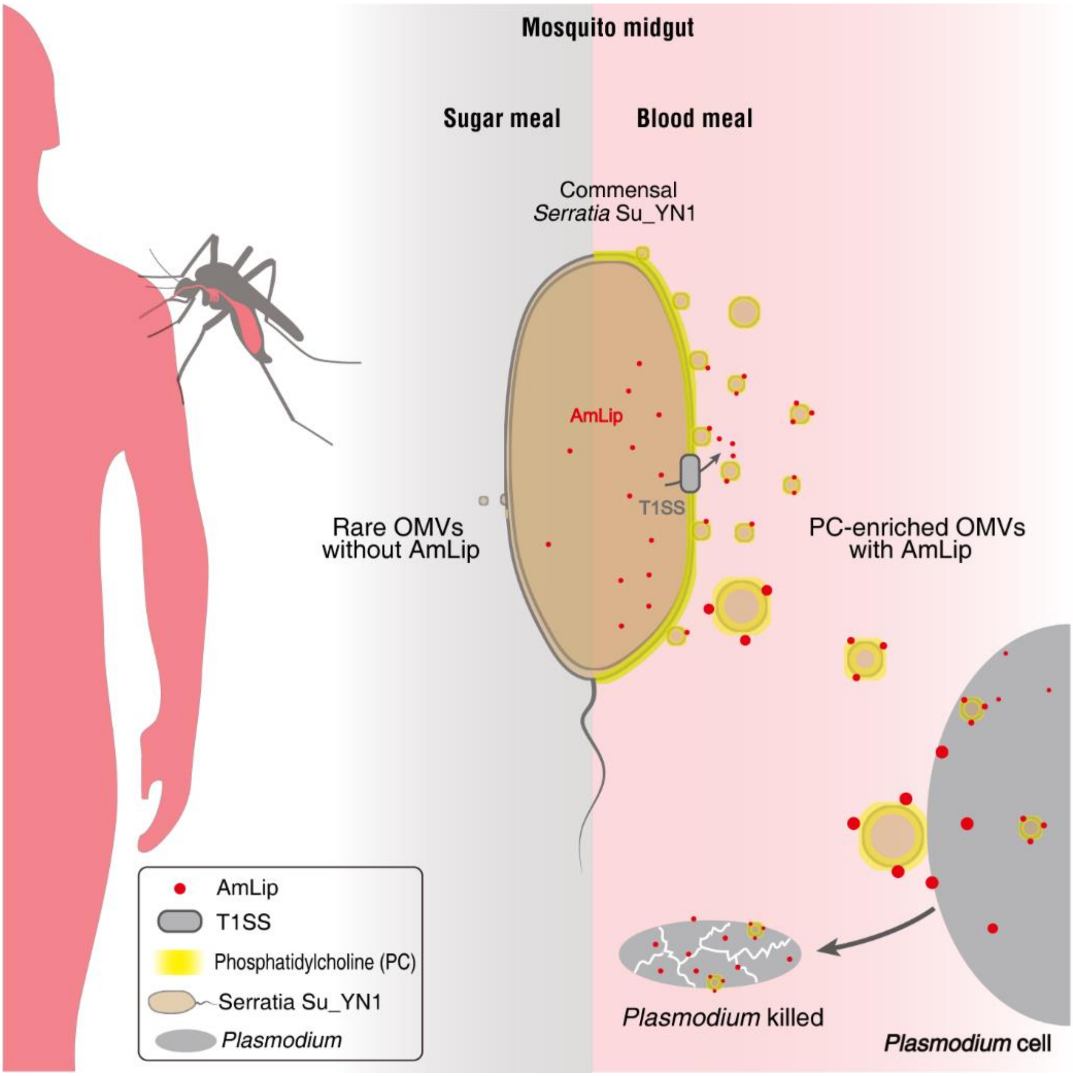

Supplementary Figure 16 | A schematic model of Su_YN1 OMVs delivering the lipase protein AmLip to *Plasmodium* parasites for targeted lysis.

After a blood meal, the symbiotic bacterium *Serratia ureilytica* Su_YN1 produces specialized outer membrane vesicles (OMVs) enriched with phosphatidylcholine (PC) in the mosquito midgut. Meanwhile, the blood meal induces Su_YN1 to secrete the anti-*Plasmodium* lipase AmLip, which is preferentially loaded onto the OMV surface after its secretion via the type I secretion system (T1SS). *Plasmodium* parasites selectively internalize these PC-enriched OMVs through the PC scavenging pathway, ultimately leading to targeted killing of the *Plasmodium* parasites by AmLip.

Uncropped scans of blots and gels of Supplementary Information

Uncropped gel showing Coomassie blue staining of Supplementary Fig.1c

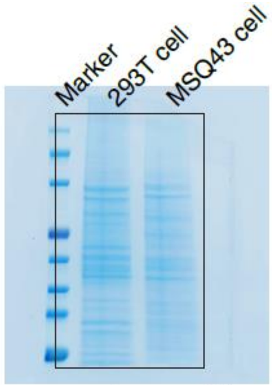

Coomassie blue staining

As loading control of Supplementary Fig.1c

Unprocessed Western blot images in Supplementary Fig.1c

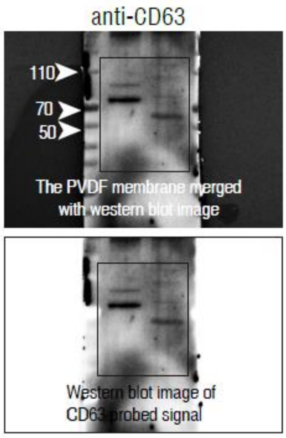

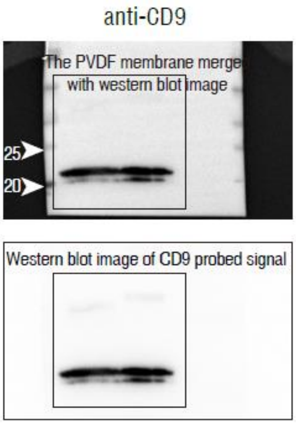

Uncropped gel showing Coomassie blue staining of Supplementary Fig.4b.

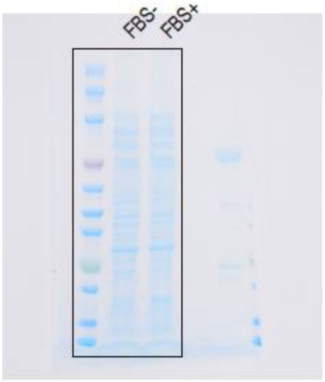

As loading control of Fig2a, total bacteria

Unprocessed Western blot images in Supplementary Fig.4e.

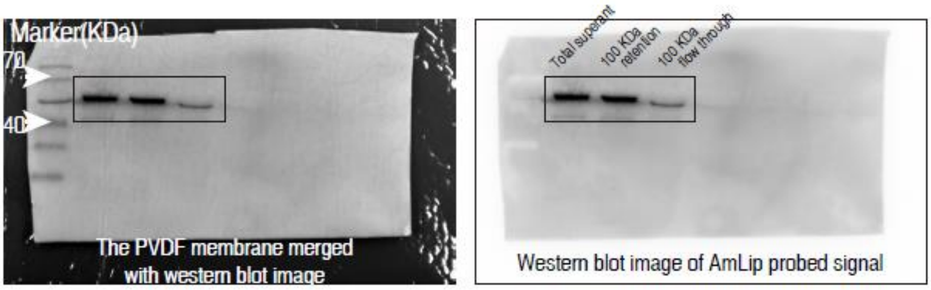

Unprocessed Western blot images in Supplementary Figure 5b.

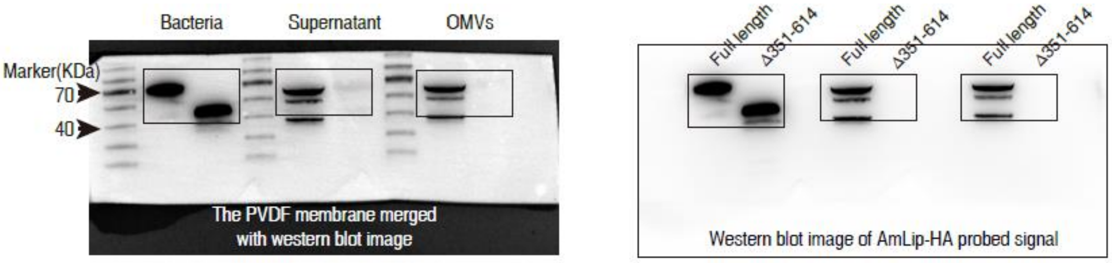

Uncropped gel showing Coomassie blue staining of Supplementary Figure 6b.

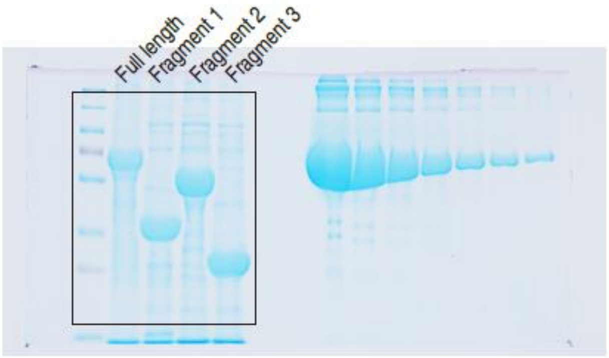

Unprocessed Western blot images in Supplementary Figure 6d.

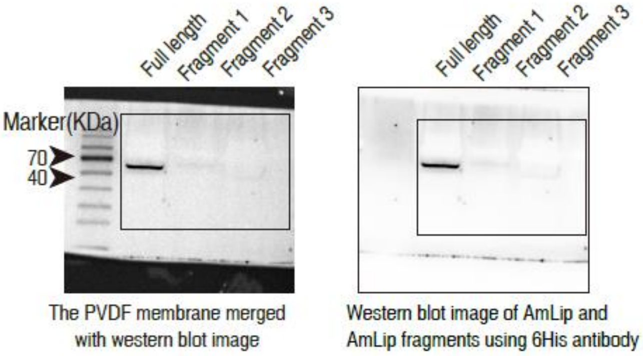

Supplement: Supplementary file 1 — Supplemental Information [file 41467_2023_40887_MOESM1_ESM.pdf]
