# Supplementary material for: Outer membrane vesicles from a mosquito commensal mediate targeted killing of Plasmodium parasites via the phosphatidylcholine scavenging pathway
Source: Nat Commun. 2023 Aug 24;14:5157. doi: 10.1038/s41467-023-40887-6 (PMC10449815; doi:10.1038/s41467-023-40887-6)
Supplement: Supplementary file 3 — Description of additional supplementary files [file 41467_2023_40887_MOESM3_ESM.docx]

**Description of additional supplementary files**

**Supplementary Movies**

**Supplementary Movies 1.**

Gliding motility tracking in Matrigel matrix of ookinetes of control group, related to Figure 3d.

**Supplementary Movies 2.**

Gliding motility tracking in Matrigel matrix of ookinetes pre-treated with 100μg /ml Su_YN1 OMVs for 5 minutes, related to Figure 3d.

**Supplementary Movies 3.**

Living tracking of *Pf* 3D7 asexual parasites co-incubated with 100μg/ml OMVs of wildtype Su_YN1 strain, related to Figure 4.

**Supplementary Movies 4.**

Living tracking of *Pf* 3D7 asexual parasites co-incubated with 100μg /ml OMVs of Δ*AmLip* Su_YN1 strain, related to Figure 4.

**Supplementary Data**

**Supplementary data 1. Proteomic analysis of Su_YN1 OMVs.**

List of LC-MSMS proteomic analysis of purified OMVs from Su_YN1 cultured in RPMI 1640 with 10% FBS.

**Supplementary data 2.** **Related to Figure 4b**.

Lipid profiles of Su_YN1 OMVs cultured with or without FBS. Su_YN1 FBS- OMVs (cultured in RPMI 1640 without FBS), FBS+ OMVs (cultured in RPMI 1640 with FBS) were purified and total lipids were extracted for lipidome analysis.

**Supplementary data 3. Related to Supplementary Figure 13a, b.**

Lipid profiles of OMVs from various resources. Su_YN1 FBS+ OMVs (cultured in RPMI 1640 with FBS), FBS- OMVs (cultured in RPMI 1640 without FBS), Su_YN1 LB OMVs (cultured in LB) and *E. coli* K12 OMVs (cultured in LB) were purified and total lipids were extracted for lipidome analysis.
